# Biosorption of Zinc from Aqueous Solution Using Chemically Treated Rice Husk

**DOI:** 10.1155/2013/365163

**Published:** 2013-06-11

**Authors:** Ying Zhang, Ru Zheng, Jiaying Zhao, Yingchao Zhang, Po-keung Wong, Fang Ma

**Affiliations:** ^1^School of Resource and Environment, Northeast Agricultural University, Harbin 150030, China; ^2^State Key Laboratory of Urban Water Resource and Environment, Harbin Institute of Technology, Harbin 150090, China; ^3^School of Life Sciences, The Chinese University of Hong Kong, Shatin, NT, Hong Kong

## Abstract

In this study, adsorption of zinc onto the adsorbent (untreated rice husk and NaOH-treated rice husk) was examined. During the removal process, batch technique was used, and the effects of pH and contact time were investigated. Langmuir isotherm was applied in order to determine the efficiency of NaOH-treated rice husk used as an adsorbent. The zinc adsorption was fast, and equilibrium was attained within 30 min. The maximum removal ratios of zinc for untreated rice husk and NaOH-treated rice husk after 1.5 h were 52.3% and 95.2%, respectively, with initial zinc concentration of 25 mg/L and optimum pH of 4.0. Data obtained from batch adsorption experiments fitted well with the Langmuir isotherm model. Maximum adsorption capacity of zinc onto untreated rice husk and NaOH-treated rice husk was 12.41 mg/g, and 20.08 mg/g respectively, at adsorbent dosage of 1 g/L at 25°C. The nature of functional groups (i.e., amino, carboxyl, and hydroxyl) and metal ion interactions was examined by the FT-IR technique. It was concluded that the NaOH-treated rice husk had stronger adsorption capacity for Zn^2+^ compared with the untreated rice husk. The NaOH-treated rice husk is an inexpensive and environmentally friendly adsorbent for Zn^2+^ removal from aqueous solutions.

## 1. Introduction 

The environmental pollution due to toxic heavy metals is spreading through the world along with industrial progress [1]. Heavy metals are major pollutants in marine, surface water and even treated wastewaters. The specific problem associated with heavy metals in the environment is their accumulation in the food chain and their persistence in nature [2]. Zinc is an ubiquitous metal ion in soil and aquatic environments. At background levels, it is an important nutrient, but at elevated concentrations due to anthropogenic processes, zinc becomes toxic. Zn^2+^ being in the list of priority pollutants proposed by Environmental Protection Agency (EPA) can give rise to serious poisoning cases. The main symptoms of zinc poisoning are dehydration, electrolyte imbalance, stomachache, nausea, dizziness, and incoordination in muscles [3]. Several different conventional methods applied to remove excessive heavy metals from aqueous solutions include chemical or electrochemical precipitation [4], ion exchange [5], adsorption on minerals, and reverse osmosis [6]. Among them, adsorption receives considerable interest in heavy metal removal. Active carbon adsorption was considered to be common and effective, but the cost is high [7]. For this reason, many studies have been carried out in order to find out effective and low-cost adsorbents. Different adsorbents are used in zinc removal such as chitosan and wheat shell [8, 9]. Rice husk (as an agricultural waste) has a large quantity of production and is evaluated as an appropriate adsorbent due to its low-cost and high removal efficiency.

The purpose of this work was to understand the adsorption mechanism of zinc from aqueous solution by using untreated rice husk and NaOH-treated rice husk. The kinetics and adsorption equilibrium of zinc onto NaOH-treated rice husk were obtained from batch experiments. The FT-IR spectrum of the adsorbent was analyzed.

## 2. Case Studies

### 2.1. Materials and Methods

The adsorbent rice husk used in this work was obtained from Harbin Kaohezhai Farm. It was washed several times with distilled water to eliminate the nutrimental materials and impurities. It was oven dried at 60°C until a constant weight was attained. In order to investigate the effect of functional groups on the surface of rice husk, it was treated with NaOH (0.1 mol/L) according to the reported method [10]. Rice husk (100 g) was maintained for 24 h in 500 mL NaOH (0.1 mol/L) solution with a stirring speed of 150 rpm and at ambient temperature. After decantation and filtration of the solution, the NaOH-treated rice husk was obtained, and then this adsorbent was dried once again. The surface morphology of NaOH-treatedrice husk was observed by using a Scanning Electron Microscope (Model S-3400N, HITACHI). FT-IR spectrum of NaOH-treated rice husk and NaOH-treated rice husk loaded with Zn^2+^ was examined by Fourier Transform Infrared Spectrophotometer (NicoletMagne 750). 

### 2.2. Chemicals

The Zn^2+^ stock solution used in this study was prepared by dissolving an accurate quantity of ZnCl_2_ with deionized water. All chemicals used in this study were of analytical grade, and solutions were prepared using deionized and distilled water. Standard solution of Zn^2+^ (1000 mg/L) for flame atomic adsorption spectrometry was obtained from Beijing NCS Analytical Instruments Co. Ltd. In order to adjust the pH, 0.1 mol/L HCl and NaOH solutions were used. 

### 2.3. Effect of Contact Time on Zn^2+^ Adsorption

Kinetic experiments were performed in continuously stirred flasks containing 100 mL Zn^2+^ solutions (concentrations of 25 mg/L) and 0.1 g of untreated rice husk or NaOH-treated rice husk from 0 to 150 min at 25°C and 150 rpm. Subsequently, the mixture was filtered using an acid-cleaned 0.45 *μ*m Millipore filter, and the concentration of Zn^2+^ in the filtrate was analyzed by flame atomic absorption spectrometry (Model AA6800, Shimadzu, Japan). The experiment was repeated three times for each condition, and the average of the three trials was determined. The relative standard deviation (RSD) for determination of Zn^2+^ using this method was approximately 5%.

The kinetics of Zn^2+^ adsorption was evaluated by applying two common models: (1) the pseudo-first-order kinetic model [11] and (2) the pseudo-second-order kinetic model [12].

The pseudo-first-order kinetic model assumes that the uptake rate of Zn^2+^ with time is directly proportional to the amount of available active site on the adsorbent surface. The general equation is expressed as

(1) $$\begin{array}{c} \frac{dq_{t}}{dt}=K_{1}\left(q_{e}-q_{t}\right), \end{array}$$

where *q*_*e*_ and *q*_*t*_ are the uptake amount (mg/g) at equilibrium and time *t*, respectively, and *K*_1_ is the pseudo-first-order adsorption rate constant (min^−1^). Integrating equation (1) for the boundary condition *t* = 0 to *t* = *t* and *q*_*t*_ = 0 to *q*_*t*_ = *q*_*t*_, the linear form of (1) becomes

(2) $$\begin{array}{c} \log\left(q_{e}-q_{t}\right)=\log\;q_{e}-\frac{K_{1}t}{2.303}. \end{array}$$

The values of adsorption rate constant were determined from the plot of log⁡ (*q*_*e*_ − *q*_*t*_) against *t* (not shown) and (2). The pseudo-second-order kinetic model assumes that chemical adsorption can be the rate limiting stage involving valence forces through sharing or exchange of electrons between adsorbent and adsorbate. The pseudo-second-order kinetic equation and the linear form are

(3) $$\begin{array}{c} \frac{dq_{t}}{dt}=K_{2}{\left(q_{e}-q_{t}\right)}^{2},\\ \frac{t}{q_{t}}=\frac{1}{K_{2}q_{e}^{2}}+\frac{t}{q_{e}}, \end{array}$$

where *K*_2_ is the pseudo second-order adsorption rate constant (g/mg·min). The initial uptake rate can be obtained as *q*_*e*_/*t* approaches zero:

(4) $$\begin{array}{c} h_{0}=K_{2}q_{e}^{2}, \end{array}$$

where *h*_0_ is the initial adsorption rate (mg/g·min). The rate constant and correlation coefficient can be calculated based on the plot (not shown) of *t*/*q*_*t*_ versus *t* for Zn^2+^ adsorption.

### 2.4. Effect of pH on Zn^2+^ Adsorption

A series of experiments, with Zn^2+^ solutions, were conducted under different pH values to investigate the effect of pH on the adsorption. The pH was primarily adjusted to a designated value, ranging from 1.5 to 6.0 with 0.1 mol/L of HCl or NaOH. The 100 mL of 25 mg/L Zn^2+^ solution was then poured into a 200 mL flask with a stopper. The sample of untreated rice husk or NaOH-treated rice husk of 0.1 g was then added to the solution, and the mixture was shaken in a temperature-controlled shaker for 4 h at 25°C and 150 rpm. The mixture was filtered, and the concentration of Zn^2+^ in the filtrate was determined by flame atomic absorption spectrometry.

### 2.5. Adsorption Equilibrium

For equilibrium studies, the concentrations of Zn^2+^ solutions varied from 10 mg/L to 100 mg/L, and, adsorbent (NaOH-treated rice husk) dosage was 1 g/L. The pH was adjusted to 3.5 (the optimum pH value) by using 0.1 mol/L of HCl or NaOH applied hourly, throughout the experiment. The mixtures were shaken for 4 h at 25°C and 150 rpm. And then they were filtered, and the concentration of Zn^2+^ in the filtrate was determined by flame atomic absorption spectrometry.

Adsorption isotherms described the adsorption process and the interaction between adsorbates and adsorbents. It is important to establish the most acceptable correlations for the batch equilibrium data for analysis and design of adsorption systems. The Langmuir and Freundlich models are the most frequently used to describe the equilibrium data of adsorption. The expressions of Langmuir equation and the linear form are

(5) $$\begin{array}{c} q_{e}=\frac{q_{m}k_{a}C_{e}}{\left(1+k_{a}C_{e}\right)},\\ \frac{C_{e}}{q_{e}}=\frac{1}{q_{m}k_{a}}+\frac{C_{e}}{q_{m}}, \end{array}$$

where *C*_*e*_ is the equilibrium concentration of Cu(II) in solutions (mg/L), *q*_*m*_ is the maximum uptake amount per g of adsorbent (mg/g), and *k*_*a*_ is the Langmuir constant related to binding energy of the sorption system (L/mg). The Langmuir parameters, *q*_*m*_ and *k*_*a*_, were calculated from the linear plots of *C*_*e*_/*q*_*e*_ against *C*_*e*_ (not shown).

The Freundlich isotherm can be described as follows:

(6) $$\begin{array}{c} q_{e}=K_{F}C_{e}^{1/n}. \end{array}$$

The linear form of the Freundlich isotherm is given by

(7) $$\begin{array}{c} \log\;q_{e}=\log\;K_{F}+\left(\frac{1}{n}\right)\log\;C_{e}, \end{array}$$

where *K*_*F*_ is the Freundlich constant indicative of the relative adsorption capacity of the adsorbent and the constant 1/*n* indicates the adsorption intensity.

The percentage removal of Zn^2+^ and equilibrium adsorption amount of Zn^2+^, *q*_*e*_ (mg/g), were calculated by using the following relationships:

(8) $$\begin{array}{c} \text{percentage}\;\text{removal}\;\text{of}\;\text{metal}\;\text{ions}=\frac{100\left(C_{0}-C_{e}\right)}{C_{0}}, \end{array}$$

adsorption amount of Zn^2+^ per gram of adsorbent (mg/g),

(9) $$\begin{array}{c} q_{e}=\;\frac{\left(C_{0}-C_{e}\right)V}{w}, \end{array}$$

where *C*_0_ is the initial concentration of Zn^2+^ (mg/L), *C*_*e*_ is the equilibrium concentration of Zn^2+^ (mg/L), *V* is the volume of the solution (L), and *w* is the mass of the adsorbent (g). 

## 3. Results and Discussion

### 3.1. Effect of pH on Zn^2+^ Adsorption

**Figure 1:**
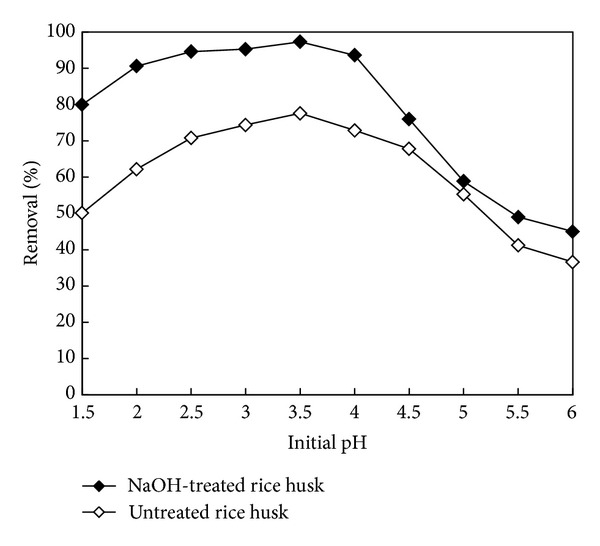
Effect of pH on the removal of Zn^2+^ onto untreated rice husk and NaOH-treated rice husk (Zn^2+^ concentration: 25 mg/L; adsorbent dosage of 0.1 g/L, at 25°C).

Many studies have shown that pH is an important factor affecting absorption of heavy metals [13, 14]. The change in initial pH affects the adsorptive process through dissociation of functional groups on the active sites on the surface of the adsorbent.  Figure 1 shows the effect of pH on the removal of Zn^2+^ onto adsorbents from aqueous solution. It was observed that with the increase of initial pH, the removal efficiency of Zn^2+^ increased firstly but decreased after attaining the pH of 4.0. The maximum removal was 97.3% and 77.6% at pH 3.5 by NaOH-treated rice husk and untreated rice husk, respectively. The change of Zn^2+^ removal was not obvious between pH 2.5 and pH 4. Absorption increased with increasing solution pH since more metal binding sites could be exposed with negative charges, with subsequent attraction of metal ions with positive charges and absorption occurring onto the cell surface [15]. This phenomenon is also attributed to the fact that substantial hydrogen ions compete for vacant adsorption sites of adsorbents at lower pH values. A decrease in removal of Zn^2+^ by adsorbent was noticed at pH values above 4.0. With the increase of initial pH above pH 4, there were more amounts of negative ions and the Zn^2+^ was surrounded by anions. It is difficult to combine with the adsorption sites on adsorbents surface of negative charge. Experiments were carried out up to pH value of 6.0 because metal precipitation occurred at higher pH values and interfered with the accumulation or biomass deterioration [16].

### 3.2. Effect of Contact Time

**Figure 2:**
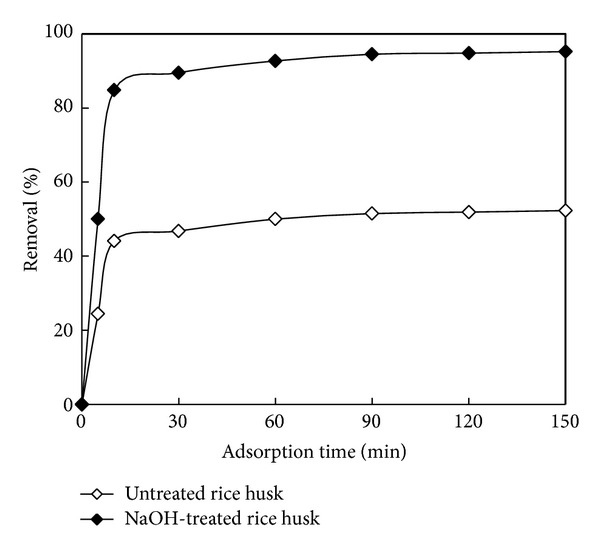
Kinetics of Zn^2+^ adsorption by untreated rice husk and NaOH-treated rice husk (Zn^2+^ concentration: 25 mg/L; adsorbent dosage of 1 g/L, at 25°C, pH 3.5).

Figure 2 shows the effect of contact time on the extent of adsorption of Zn^2+^ by adsorbent. The percentage removal of Zn^2+^ for untreated rice husk and NaOH-treated rice husk after 150 min was 52.3% and 95.2%, respectively, under the same experimental conditions. The percentage removal of Zn^2+^ was rather fast in the previous 10 minutes, but then the removal significantly decreased and eventually reached a plateau after 30 min. This phenomenon may be related to the vacant adsorption sites on adsorbent surface. During the initial stage of sorption, a large number of vacant surface sites are available for adsorption. After lapse of time, the remaining vacant surface sites can be occupied difficultly due to repulsive forces between the solute molecules on adsorbent surface and the bulk phase [17]. And there were fewer vacant surface active sites that could be occupied by metal ions after lapse of time. The data obtained from this experiment was further used successfully to evaluate the kinetics of the adsorption process.

Adsorption kinetics, which is one of the important characteristics defining the efficiency of sorption, described the solute uptake rate. The rate constants and correlation coefficients of kinetics for the Zn^2+^ adsorption are presented in Table 1. Results show that the adsorption process of untreated rice husk and NaOH-treated rice husk both fitted better with the pseudo-second-order model with *R* ^2^ of 0.9974 and 0.9912, respectively. The adsorption amount and rate of NaOH-treated rice husk were significant compared with untreated rice husk significantly. 

### 3.3. Adsorption Isotherm for NaOH-Treated Rice Husk

**Figure 3:**
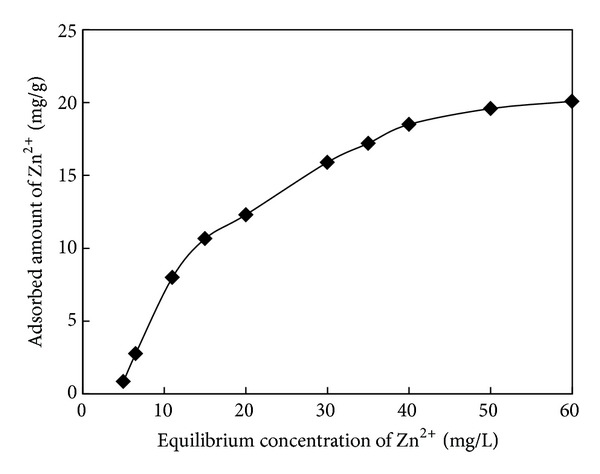
Adsorption isotherms of Zn^2+^ by NaOH-treated rice husk (adsorbent dosage of 1 g/L, at 25°C, pH 3.5).

The equilibrium adsorption isotherm is one of the most important data to understand the adsorption mechanisms. As indicated in Figure 3, the isothermal curve of Zn^2+^ for NaOH-treated rice husk showed a typical Langmuir-type pattern. The relationship between the reciprocal of the amount of Zn^2+^ adsorbed on NaOH-treated rice husk and the reciprocal of the equilibrium concentration of Zn^2+^ in the solution was linear. Adsorption capacity (*q*_*e*_) was determined based on the adsorption isotherms of untreated rice husk and NaOH-treated rice husk. 

The parameters of adsorption isotherms for untreated rice husk and NaOH-treated rice husk are shown in Table 2. Results show that the adsorption process fitted well with the Langmuir isotherm model with correlation coefficient (*R* ^2^) of 0.9976 and 0.9985 for untreated rice husk and NaOH-treated rice husk, respectively. Within the Langmuir model, the saturation adsorption capacity *q*_*m*_ is supposed to coincide with saturation of a fixed number of identical surface sites and as such, it should logically be independent of temperature [21]. Maximum adsorption capacity of Zn^2+^ onto NaOH-treated rice husk had been improved obviously compared with untreated rice husk. The results obtained from this study were found to be higher than those of many corresponding biosorbents reported in the literature (Table 3) [18–20, 22].

### 3.4. Characterization of NaOH-Treated Rice Husk

**Figure 4:**
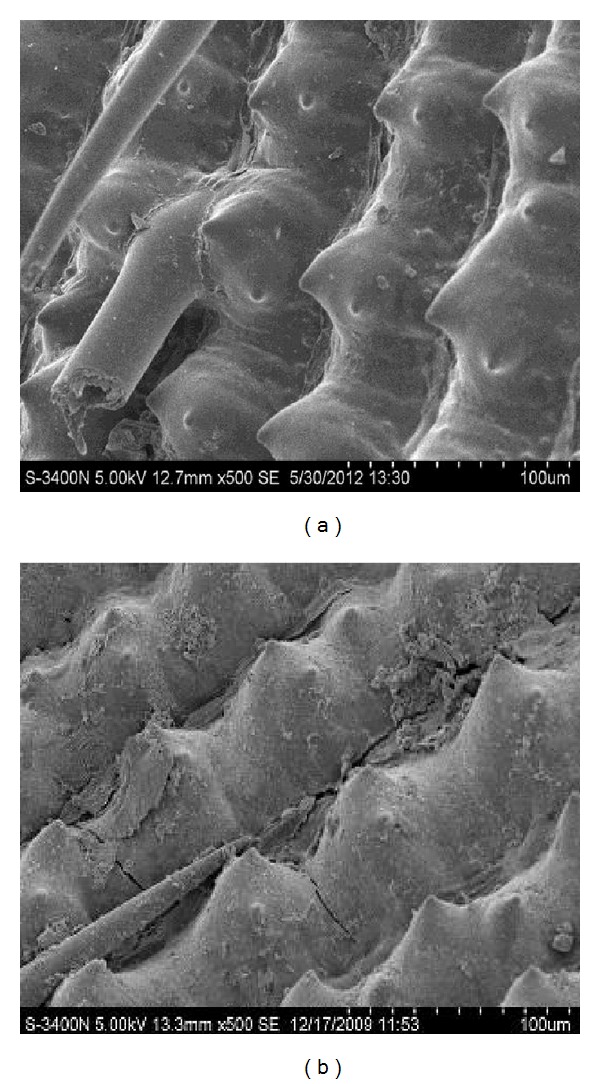
Scanning electron micrographs of (a) untreated rice husk and (b) NaOH-treated rice husk.

Scanning electron micrographs of untreated rice husk and NaOH-treated rice husk are shown in Figure 4. At low power electron microscope, some regular and isscross framework constructions can be observed on the surface of the untreated rice husk in Figure 4(a). Embossments approximated cone and epidermal villi like needle can be seen between the two lines of framework. The surface morphological structure of NaOH-treated rice husk is shown in Figure 4(b). There were a lot of “point spots” on cell wall and regular and criss-cross skeleton structure. The lipid, protein, and soluble polysaccharide in the rice husk were dissolved as the rice husk was treated with NaOH. The surface structure on the cell was loose, and the physical and chemical property of rice husk was improved. This morphological structure of NaOH-treated rice husk is conductive to the uptake of metal ions. And the structure of the adsorbent meant that the NaOH-treated rice husk cell wall plays an important role in the Zn^2+^ adsorption process. 

**Figure 5:**
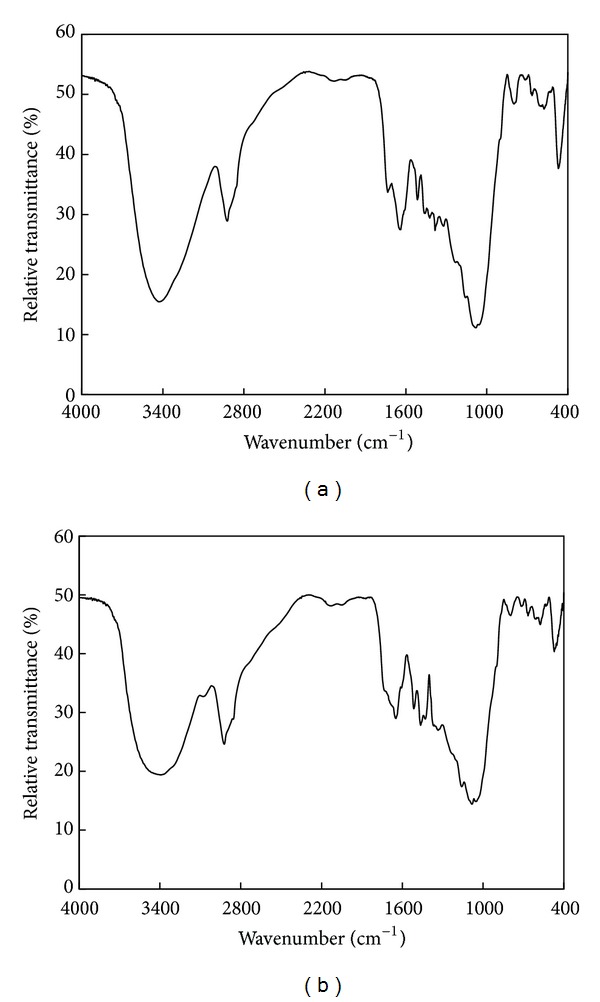
FT-IR spectrum of (a) NaOH-treated rice husk and (b) NaOH-treated rice husk loaded with Zn^2+^.

The FT-IR absorbance spectrum of NaOH-treated rice husk and NaOH-treated rice husk loaded with Zn^2+^ is shown in Figure 5. The peaks at 3400.27 cm^−1^, 1406.01 cm^−1^, and 1033.77 cm^−1^ were due to amino, carboxyl, and hydroxyl groups existence on the cell wall of NaOH-treated rice husk. It can be seen that after adsorbing Zn^2+^, the peaks at 3400.27 cm^−1^, 1406.01 cm^−1^, and 1033.77 cm^−1^ reduced to 3398.34 cm^−1^, 1386.72 cm^−1^ and 1041.49 cm^−1^, respectively, which suggested that amino, carboxyl, and hydroxyl groups could combine intensively with Zn^2+^. The analysis of the FT-IR spectrum showed the presence of ionisable functional groups (i.e., amino, carboxyl, and hydroxyl) able to interact with metal ions. Kapoor and Viraraghavan had shown that esterification of carboxyl groups or methylation of amino groups of Aspergillus niger biomass severely inhibited the Lead biosorption [23]. Bai and Abraham had revealed that acetylations of amino and hydroxyl groups of *Rhizopus nigricans* biomass obviously reduced the chromium absorption [24]. These facts indicated the important role played by carboxyl, amino, and hydroxyl groups in the absorption of heavy metals. It suggested that ion exchange should be the principal mechanism for Zn^2+^ biosorption by using NaOH-treated rice husk.

## 4. Conclusion

The adsorption properties of Zn^2+^ by untreated rice husk and NaOH-treated rice husk were examined in this experiment. The saturated adsorption amount of Zn^2+^ on NaOH-treated rice husk was 20.08 mg/g. The maximum removal of Zn^2+^ for untreated rice husk and NaOH-treated rice husk after 150 min was 52.3% and 95.2% at optimum pH of 3.5, respectively. The data of FT-IR spectrum confirmed the presence of ionisable functional groups (i.e., amino, carboxyl, and hydroxyl), and they were able to interact with Zn^2+^. This study shows that the NaOH-treated rice husk is an inexpensive and environmentally friendly adsorbent for Zn^2+^ removal from wastewater.

## Figures and Tables

**Table 1:** Kinetic parameters for the adsorption of Zn^2+^ by untreated rice husk and NaOH-treated rice husk.

Adsorbent	Pseudo-first-order model			Pseudo-second-order model			
	*K*_1_ (min^−1^)	*q*_*e*_ (mg/g)	*R* ^2^	*K*_2_ (g/mg·min)	*q*_*e*_ (mg/g)	*h*_0_ (mg/g·min)	*R* ^2^
Untreated rice husk	0.02	7.59	0.9507	0.0008	10.27	0.0844	0.9974
NaOH-treated rice husk	0.03	16.90	0.9719	0.0017	22.83	0.8861	0.9912

**Table 2:** Isotherm parameters for the adsorption of Zn^2+^ by untreated rice husk and NaOH-treated rice husk.

Adsorbent	Freundlich model			Langmuir model		
	*K*_*F*_	1/*n*	*R* ^2^	*k*_*a*_ (L/mg)	*q*_*m*_ (mg/g)	*R* ^2^
Untreated rice husk	0.47	0.36	0.9303	12.84	12.41	0.9976
NaOH-treated rice husk	2.21	0.80	0.9732	7.32	20.08	0.9985

**Table 3:** Adsorption of Zn^2+^ from the literature by various adsorbents.

Adsorbents	Adsorption capacity (mg/g)	Reference
Activated carbon obtained from agricultural by-products	6.65	Ferro-García et al. [18]
Oxidised jute	8.02	Shukla and Pai [19]
Neem bark	13.29	Bhattacharya et al. [20]
Black gram husk	13.45	Saeed et al. [9]
Clarified sludge	15.53	Bhattacharya et al. [20]
NaOH-treated rice husk	20.08	This study
